# Signatures of balancing selection in toll-like receptor (TLRs) genes – novel insights from a free-living rodent

**DOI:** 10.1038/s41598-018-26672-2

**Published:** 2018-05-30

**Authors:** Agnieszka Kloch, Marius A. Wenzel, Dominik R. Laetsch, Olek Michalski, Anna Bajer, Jerzy M. Behnke, Renata Welc-Falęciak, Stuart B. Piertney

**Affiliations:** 1 University of Warsaw, Department of Ecology, Faculty of Biology, Biological and Chemical Research Centre, ul. Zwirki i Wigury 101, 02-089 Warszawa, Poland; 2 University of Aberdeen, Institute of Biological and Environmental Sciences, Tillydrone Avenue, Aberdeen, AB24 2TZ UK; 3 The James Hutton Institute, Dundee, DD2 5DA United Kingdom; 4 University of Edinburgh, Institute of Evolutionary Biology, Edinburgh, EH9 3JT UK; 5 Data Analysis & Visualisation, ul. Przy Agorze 5a m. 98, 01-960 Warszawa, Poland; 6 University of Warsaw, Department of Parasitology, Faculty of Biology, ul. Miecznikowa 1, 02-096 Warszawa, Poland; 7 School of Life Sciences, University of Nottingham, Nottinghamshire NG7, United Kingdom

**Keywords:** Evolutionary ecology, Molecular evolution

## Abstract

Selective pressure from pathogens is considered a key selective force driving the evolution of components of the immune system. Since single components of the immune system may interact with many pathogens, and single pathogens may be recognized by multiple components of the immune system, gaining a better understanding of the mechanisms of parasite-driven selection requires the study of multiple genes and pathogens. Toll-like receptors (TLRs) are a large gene family that code for antigen-presenting components of the innate immune response. In the present paper we characterize polymorphism and signatures of selection in seven TLRs in free-living bank voles *Myodes glareolus*. We report the first evidence of balancing selection in several TLR genes, supported by positive values of Fu and Li’s D* in TLR2 and TLR5, and positive values of Tajima’s D in LRR regions within TLR1 and TLR2. We further found significant associations between amino-acid alleles of TLR1 and TLR5 and susceptibility to infection with the blood pathogen *Bartonella*. Interestingly, selection patterns in TLRs presenting virus-derived motifs (TLR7 and TLR9) differed considerably from those interacting with bacterial PAMPs. In contrast to the highly variable TLRs presenting bacterial motifs, TLR7 and TLR9 had low polymorphism and displayed signatures of directional selection. These findings suggest different functional responses across the TLR gene family and highlight the complexity of parasite-driven selection.

## Introduction

Host-pathogen relationships have long been in the focus of evolutionary biology as a prominent example of an evolutionary arms race. In the wild, animals are exposed to a spectrum of pathogens, and each pathogen employs different mechanisms to escape from host defence. As a result, components of the immune system are expected to evolve under parasite-driven selection. The strength and direction of this selection depends on the pathogen repertoire, as a single component of the immune system may interact with many pathogens, while single pathogens may be recognized by multiple components of the immune system. Given the complexity of these interactions, understanding the mechanisms of pathogen resistance in the wild requires the study of multiple loci and multiple pathogens^1^.

However, much of what we know about mechanisms of parasite-driven selection has been based on a single locus, namely major histocompatibility complex (MHC). MHC constitutes part of the adaptive immune response, which provides specific but delayed reaction to an infection. Moreover, most variance among hosts in their resistance against pathogens may be explained by factors other than MHC repertoire^2^, and other immunity genes have been shown to play a crucial role in response against helminth infections in free-living vertebrates (e.g.^3–5^).

Toll like receptors (TLRs) are the key component of the innate immunity. They recognize pathogen-associated molecular patterns (PAMP) derived from microorganisms such as viruses, bacteria, protozoa, and fungi. After binding to a motif, TLR molecule triggers inflammation and activates signalling pathways which initiate later stages of the immune response, including the adaptive response. Clinical studies have shown that polymorphism within TLR genes, particularly in the part of the molecule interacting with PAMP, affects susceptibility to diseases in humans – for instance, a single nucleotide polymorphism (SNP) in the TLR2 gene has been attributed to susceptibility to tuberculosis, cytomeglaovirus, and borreliosis, whereas SNPs associated with susceptibility to hepatitis C virus have been identified in TLR7 (see the work reviewed in^6^). Associations between TLR variants and susceptibility have also recently been found in TLR2 and TLR4^4,7^ in free living species.

Selection acting on TLR genes has seldom been studied in a microevolutionary context, and numerous studies that have focused on macro-evolutionary processes have provided no clear picture. Positive selection has been reported in primates^8,9^, and across several mammalian taxa^10^. In birds, TLR evolution was found to be dominated by stabilizing selection and slow rates of non-synonymous substitutions^11^. In two closely related newt species, purifying selection was observed over a long time scale but evidence for positive selection was found on a medium timescale^12^. Across several rodent species, signatures of positive selection were detected in PAMPs of TLR2 but the remaining part of the protein seemed to evolve under purifying selection^13^. TLRs presenting bacterial ligands tend to differ in their evolutionary pattern from TLRs interacting with viral motifs^8,14^, yet this finding was not supported in all taxa studied^10^.

Although immunity genes affected by parasite-driven selection are likely to evolve under balancing selection, to date this has only been reported in TLR1, 6, and 10 in humans, in the European population^15^. It was not observed in other populations, which authors have explained in terms of different pathogen pressures, yet the effect of pathogens was not directly included in the analysis. The effect of balancing selection on genomic regions is often subtle compared to positive selection^16^, and may be difficult to detect over a long evolutionary scale^17^. To better understand micro-evolutionary processes affecting polymorphism within TLR genes, studies on a small spatial or temporal scale are necessary. Hence, in the current paper we tested signatures of selection as well as associations between infections and genetic variants. The sites studied differ in terms of pathogen communities, and these differences are stable over time^18^. In our previous work in this study system, we found local, contrasted associations between MHC DRB beta chain variants and susceptibility to infection with the nematod*e Aspiculuris tetraptera*^19^.

## Methods

### Material and pathogen screening

DNA samples were obtained from 90 bank voles (*Myodes glareolus*) collected at two sites (Urwitałt and Pilchy, NE Poland) in a single trapping session in 2005. These individuals were previously genotyped in MHC-DRB and 6 microsatellite loci^19^. The helminth infection level was determined based on autopsy material; for details see^19^. In the present study we also considered infection with microparasites. The intestinal protist *Cryptosporidium sp*. was identified in faecal smears using the Ziehl-Nielsen staining technique^20^. The blood pathogens *Bartonella sp*. and *Babesia sp*. were identified by PCR using primers BhCS.781p and BhCS.1137n for *Bartonella sp*.^21^, and nested reaction with primers CRYPTO R and CRYPTO F^22^, and Bab1 and Bab4 for *Babesia*^23^. Sequences of the primers and reaction conditions are given in Table S1. The remaining blood pathogens – the bacteria *Haemobartonella sp*. and the protists *Trypanosoma sp*. and *Hepatozoon sp. –* were microscopically identified based on size, shape and colouration upon Giemsa-staining. The list of the identified species, along with their prevalence, is given in Table S2.

All procedures were performed in compliance with the Animal Protection Act of 1997 (Dz.U. 1997 no. 111 pos. 724), followed the guidances of the National Ethics Committee for Experimentation on Animals (Poland), and were approved by the Local Ethical Committee no. 1 in Warsaw, decision no. 280/2003.

### Library preparation

Primers amplifying toll-like receptors TLR1, TLR4, TLR5, TLR6, TLR7 and TLR9 were designed in conserved regions of aligned rodent mRNA sequences obtained from the NCBI GenBank. Despite several attempts, we were not able to design primers amplifying TLR3 and TLR8. TLR2 was amplified using two pairs of overlapping primers described in^13^.

To minimize the PCR error rate, we used high fidelity Phusion polymerase (Thermo Scientific). The PCR mix contained: 5x polymerase buffer, 200 uM of each dNTP, 0.5 uM of each primer, 0.3 U of polymerase, and 10–50 ng of genomic DNA. Details of both primers’ melting temperature and annealing time are given in Table S3.

Amplified fragments were pooled for each individual in similar quantities based on intensities of resolved bands by agarose gel electrophoresis^24^, and purified twice using CleanUp kit (Aabiot). The library was prepared using Nextera XT DNA according to the manufacturer’s protocol and sequenced in a single run on Illumina MiSeq using MiSeq Reagent Kit v3 (150 cycles) producing 2 × 75 bp reads. The quality of the read datasets was assessed using the FastQC tool (https://www.bioinformatics.babraham.ac.uk/projects/fastqc).

### Assembly and genotyping

To construct a reference for read mapping, an unannotated bank vole genome assembly was retrieved from the NCBI GenBank (PRJNA290429). Mouse transcripts (accession numbers NM_001276445, NM_011905, NM_021297, NM_016928, NM_011604, NM_001290755, NM_031178) of the studied TLR genes were mapped against the bank vole genome using the -f samse option of gmap tool (http://research-pub.gene.com/gmap)^25^. The regions against which mouse transcripts mapped were extracted with ~1000 bp offset using getfasta function in Bedtools ver. 2.25 (http://bedtools.readthedocs.io/en/latest)^26^ and used as reference in the downstream analysis.

The TLR9 locus in the bank vole genome contained two unassembled parts (-NNN-). Thus, after initial mapping as described below, we designed internal primers (TLR9_intF 5′TCTGCCCAACCTCCATACTC3′ and TLR9_intR 5′ TCTTATGGTCAGGGGTGCTC3′), which, together with the TLR9 F/R primers listed in Table S3, were used to amplify regions overlapping the -NNN- parts. The amplicons were Sanger-sequenced providing reference sequences with filled -NNN- parts that were used to construct a gap-free reference sequence for this locus.

Paired-end reads were mapped against the reference using bwa-mem ver. 0.5.9 (http://bio-bwa.sourceforge.net) with default parameters^27^. The resulting bam files were merged, and deduplicated using the Picard MarkDuplicates tool (http://broadinstitute.github.io/picard). Duplicated read-pairs constituted 87% of the reads. For the further analysis, we retained only properly mapped and paired read pairs (both reads mapped, correct insert size) with a mapping quality ≥20, which resulted in 32 213 487 reads. The high coverage obtained (~100 000×) assured high confidence of the genotyping results.

Variants were called in two-step procedure using the FreeBayes v0.9.10-3 (https://github.com/ekg/freebayes)^28^. First, we ran FreeBayes with the following parameters: minimal fraction of alternate allele of 20% (-F 0.2) as recommended^29^, minimum number of reads supporting alternate allele >2 (–min-alternate-count 2), and minimal read coverage >5 (–min-coverage 5). The results were filtered using vcffilter v.41 (https://github.com/vcflib/vcflib) specifying: QUAL/AO > 10 & DP > 10 & SAF > 0 & SAR > 0 & RPR > 0 & RPL > 0. In a second step, filtered high confidence variants were used to construct haplotypes using–hapolotype-basis-alleles function and specifying–max-complex-gap 37 (i.e. half of the read length). SNPs which were not phased by Freebayes were computationally phased using PHASE (http://stephenslab.uchicago.edu/software.html#phase)^30^ and resolved into haplotypes using a custom Python script (included in the Supplementary Materials).

The reading frame in each locus was identified based on alignment with mouse transcripts, which allowed each SNP to be classified as synonymous or non-synonymous. Additionally, in each locus we identified leucine-rich repeats (LRRs) using the LRRfinder tool (http://www.lrrfinder.com)^31^.

### Sequence variation and signatures of selection

Basic measures of sequence diversity – the number of variable sites (S), haplotype diversity (Hd), average number of nucleotide differences (k), and nucleotide diversity per site (pi) – were summarized in DnaSP v.5 (http://www.ub.edu/dnasp)^32^. Observed and expected heterozygosity and F_is_ (inbreeding coefficient) were calculated using the R package diveRsity^33^, and the F_st_ statistic was computed in Arlequin ver 3.1 (http://cmpg.unibe.ch/software/arlequin35)^34^.

In the present paper we focused on signatures of selection acting on TLR genes on a small evolutionary scale. First, we tested for deviations from Hardy-Weinberg equilibrium (HWE) using the Markov chain method in Genepop v.4 (http://kimura.univ-montp2.fr/~rousset/Genepop.htm)^35^. In DnaSP, we computed two neutrality tests based on a frequency spectrum of alleles and segregating sites: Tajima’s D^36^ and Fu and Li’s D*^37^. Tajima’s D was also calculated using a 60 bp sliding window and 20 bp step size. The size of the window corresponded to the average size of a LRR. Using mouse sequence (*Mus musculus*) as an outgroup we computed the McDonald-Kreitman (MK) test^38^, which compares the rate of synonymous and non-synonymous substitutions within and between species, and allows for the detection of selection acting on a longer time scale. Unlike Tajima’s D and Fu and Li’s D*, this test is relatively insensitive to demographic assumptions. Additionally, in Arlequin we computed the Ewens-Watterson test, which allows contemporary selection to be distinguished from past selection^39,40^.

### Associations between genetic variation and infection

We used generalized linear models to identify associations between genetic polymorphisms and susceptibility to infections. This approach is an alternative to selection tests, and detection of significant associations between genetic variants and presence of pathogens indicates contemporary parasite-driven selection operating at the studied loci^41^.

First, we tested the significance of three non-genetic variables: host sex, body mass, and sampling site, which were previously shown to influence nematode load in the studied populations^19^. For the most frequent eight pathogen species, whose overall prevalence exceeded 20% (Table S2), we fitted models with binary infection status as the dependent variable, using GLM with binomial error distribution and logit link function in R^42^. Non-genetic terms that were associated with the risk of infection with p < 0.05 (Table S4) were included as explanatory variables in the models for the respective pathogen species.

In a second step, we selected genetic variants to be included in the models. As recommended in^43^, we focused on haplotype polymorphism rather than single nucleotide polymorphism (SNP). By using haplotypes, which are combinations of all SNPs within a locus, we were able to reduce the number of explanatory variables fitted to a model without losing information about the genetic variability within the studied genes. To better account for functional rather than structural differences between variants, we translated nucletoide sequences into amino-acid sequences. Such an approach has been used in similar studies examining the functional effects of the components of the immune system on susceptibility to infections^7,44^. From here on in this paper, unless stated otherwise, we use the term “haplotype” to denote a unique amino-acid haplotype. For clarity, the amino acid haplotypes are denoted TLR*aa. After translation, only two unique amino acid sequences were found both in TLR7 and TLR9. The less frequent allele of TLR7 was present only in four individuals, and of TLR9 only in one animal, thus we were not able to test for an effect of any of these loci using GLM models.

To control for relatedness between individuals and possible population structure, we computed a pairwise relationship matrix based on six previously genotyped microsatellite loci^19^ using the irelr package for R (https://github.com/andersgs/irelr)^45^. We summarized the variation using principal component analysis (PCA). The first and second principal components (PC1 and PC2) were fitted to each model.

For each locus (TLR1, 2, 4, 6) and each pathogen, we constructed separate models with infection status as dependent variable. In each model the explanatory variables were as follows: (1) optional non-genetic terms as described above, (2) presence/absence of the most frequent haplotypes, (3) PC1 and PC2 from the relatedness matrix summarizing population structure. To avoid overfitting, we only considered haplotypes that occurred in at least 8 individuals (~10% of the studied animals) with the exception of models including *H. mixtum* and *H. glareoli*. Each of these nematodes was observed in only one of the studied sites: *H. mixtum* at the Urwitałt site, *H. glareoli* in Pilchy. This pattern is persistent over time, as reported in a longitudinal study of the rodent parasite fauna at these sites^18^. Thus, models including either *H. mixtum* or *H. glareoli* were fitted using observations from the respective sites, and to these models we fitted the 5 most frequent TLR variants from each site. To control for multiple comparisons, we adjusted the p-values of genetic terms using the false discovery rate (FDR) procedure^46^. Following^47^, we considered an adjusted p-value to be highly significant at p < 0.05, and significant at p < 0.1.

To provide a quantitative measure of the strength of the tested effects, we calculated R^2^, an effect size statistic in the rsq package for R^48^. R^2^ is a coefficient of determination that measures the proportion of the variance in the dependent variable which can be predicted from the independent variable.

Additionally, to complement the haplotype-oriented analysis, we tested the effects of SNPs on the risk of infection. Synonymous SNPs were filtered using the PLINK 1.9 tool (https://www.cog-genomics.org/plink2)^49^. We retained individuals with at least 30% of SNPs called, and SNPs that were in the Hardy-Weinberg equilibrium (P-value cutoff 0.01) with less than 10% missing data and with minor allele frequency greater than 0.01. Linkage disequilibrium among all SNP pairs was examined using squared allele-counts correlation (r^2^), and SNPs were thinned out such that r^2^ among SNP pairs within a sliding window of 50 SNPs (step-size 10 SNPs) was below 0.7. Filtered SNPs were fitted as explanatory variables to GLM models constructed in a similar manner as in the case of haplotype-based models, with pathogen presence/absence as dependent variable, and genotype at a given SNP, two PCs summarizing the relatedness among individuals, and optional non-genetic terms as explanatory variables. The effect of each SNP was tested separately for each dependent variable, with p-levels adjusted using FDR.

### Data Accessibility

Raw reads have been stored in SRA archive, biosample no. 7414489. Haplotypes are stored in GenBank (Access nos. MF471907 – MF472008). Data on infection status and genotypes of individuals are available at github.com/drowca/TLRs.

## Results

### Genetic polymorphism and test of selection

Overall, we found 179 variable sites in 15043 bp across seven TLR genes, including 177 SNPs and two indels (Table 1). A three-nucleotide deletion was detected in TLR4 in position 1830 (haplotype 8) in four heterozygous individuals. In TLR5, a two-nucleotide deletion present in 17 individuals was identified in position 247 of haplotypes 2 and 16, which resulted in a frame shift mutation and a premature stop codon in position 379.

**Table 1:** Summary of the diversity of the studied loci.

locus	length (bp)	n	%	S	S/1000 bp	h	AA	Hd	k	π × 10^3^
TLR1	1126	81	47.2	24	21.31	16	12	0.851	6.387	5.67
TLR2	2351	85	99.9	57	24.25	32	26	0.804	9.116	3.80
TLR4	2162	81	86.3	21^1^	9.71	16	11	0.771	2.708	1.25
TLR5	2260	86	86.3	39^1^	17.3	19	10	0.837	7.403	3.28
TLR6	1234	85	51.0	20	16.21	12	8	0.811	5.464	4.43
TLR7	2948	86	93.6	5	1.7	6	2	0.347	0.375	0.13
TLR9	2962	83	95.8	11	3.71	9	2	0.753	3.372	1.14

Length of the genotyped sequence in bp and % – percent of protein-coding sequence covered by genotyping are given. n – number of genotyped individuals, S – number of variable sites, h – number of haplotypes, AA – number of amino-acid alleles (unique translated sequences), Hd – haplotype diversity, k – average number of nucleotide differences, π – nucleotide diversity per site. ^1^Including an indel.

Levels of polymorphism differed considerably between the loci studied. The most polymorphic loci were TLR1 and TLR2 with over 20 variable sites per 1000 bp; in these two loci we observed the highest average number of nucleotide differences (k), and the highest nucleotide diversity per site (π) (Table 1). The number of segregating sites and the nucleotide diversity per site were much lower in TLR7 and in TLR9 than in other loci, and TLR7 had considerably lower haplotype diversity compared to other loci.

We found significant deviations from the Hardy-Weinberg equilibrium in TLR4, TLR7, and TLR9 (Table 2). The F_st_ coefficient showed no consistent pattern over loci: in TLR1, 2, 4 and 6 it was lower than 0.1, suggesting a low level of population differentiation, in contrast to TLR9, where F_st_ exceeded 0.25, indicating strong differentiation. By comparison, F_st_ was 0.072 in microsatellites, and 0.069 in MHC-DRB^19^.

**Table 2:** Genetic differences between studied sites.

locus	F_st_	F_is_	HWE		
			both sites	Urwitałt	Pilchy
TLR1	0.0998^***^	0.0428	0.099	0.132	0.154
TLR2	0.0569^***^	−0.0091	0.784	0.497	0.869
TLR4	0.0166^*^	−0.0645	0.017	0.057	0.043
TLR5	0.1174^***^	0.0197	0.958	0.729	0.992
TLR6	0.0662^***^	0.0422	0.410	0.258	0.564
TLR7	0.1069^***^	0.4315	<0.001	0.156	0.000
TLR9	0.3833^***^	0.0889	0.027	0.057	0.074

F_st_ – fixation coefficient (p-value given in a upper index, ***p < 0.001, **p < 0.1, *p < 0.05), F_is_ – inbreeding coefficient, HWE – p-value of Hardy-Weinberg equilibrium test estimated by the Markov chain method.

Tajima’s D tests were not significant for any locus, but we found significantly positive values of Fu and Li’s D* in TLR2 and TLR5 (Table 3). These two loci were also significant at p < 0.02 when Fu and Li’s test was computed with a mouse sequence as an outgroup (Table S5). The McDonald-Kreitman (MK) test revealed an excess of synonymous substitutions in TLR9 (Table 3). The Ewens-Watterson results were not significant at any locus, suggesting that the effect of long-term selection did not affect the observed homozygosity.

**Table 3:** Results of neutrality tests.

	Tajima’s D	Fu & Li D*	McDonald-Kreitman (NI)	Ewens-Watterson
TLR1	1.424^ns^	0.115^ns^	0.768^ns^	0.337
TLR2	−0.267^ns^	**1.089** ^< **0.02**^	1.469^ns^	0.996
TLR4	−0.744^ns^	−0.128^ns^	0.641^ns^	0.543
TLR5	0.254^ns^	**1.841** ^< **0.02**^	1.070^ns^	0.633
TLR6	1.331^ns^	1.297^ns^	0.609^ns^	0.260
TLR7	−1.118^ns^	−0.136^ns^	2.193^ns^	0.845
TLR9	1.813^ns^	0.678^ns^	**0.138** ^**0.031**^	0.290

Coefficients given for each test, with the p-values in superscript. For EW test, only p-values are presented. Significant variables are marked in bold.

**Figure 1:**
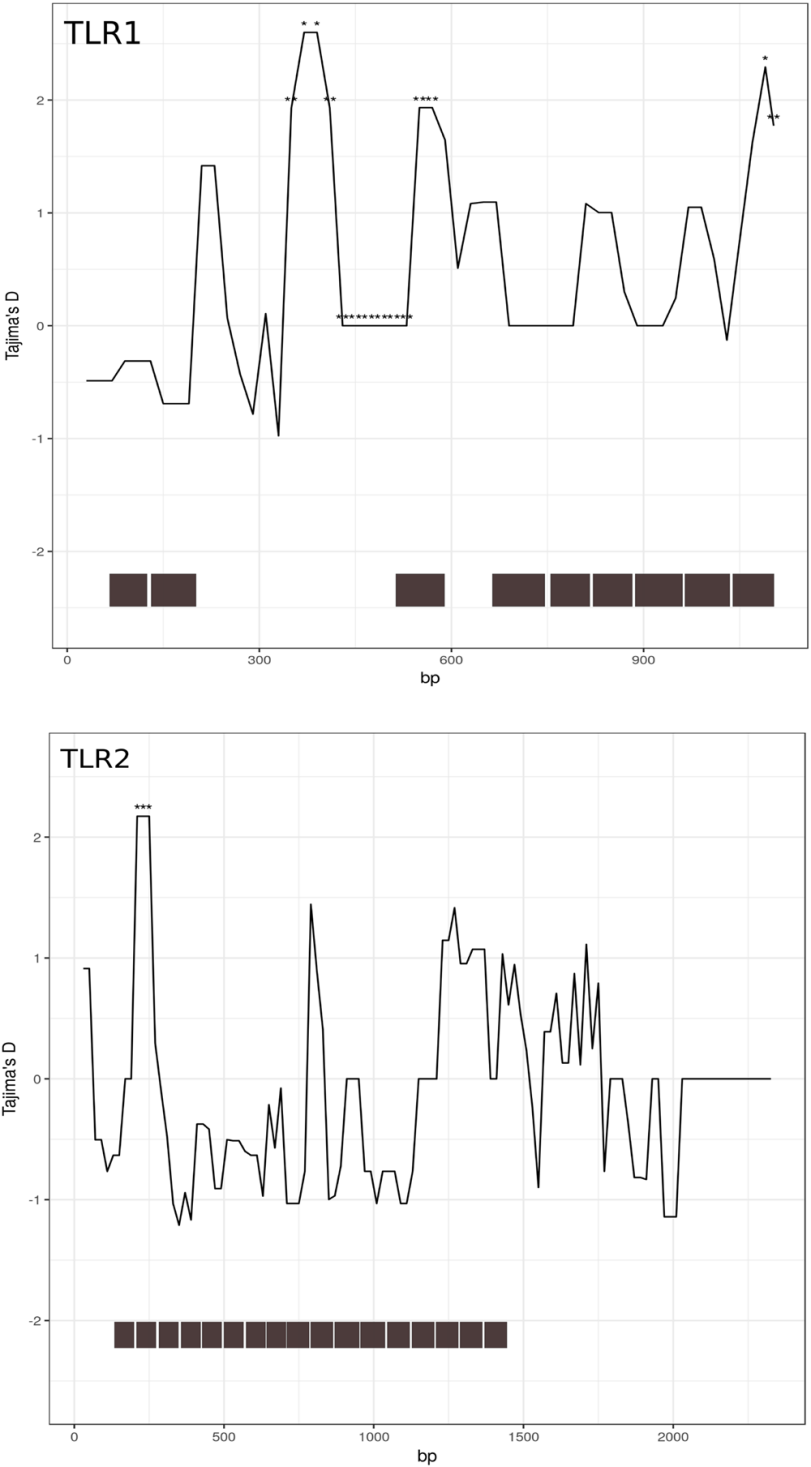
Sliding window Tajima’s D for TLR1 and TLR2. Stars indicate regions where D was significant at p < 0.05. Grey bars below the graph represent location of LRRs.

The sliding-window Tajima’s test revealed regions of positive D (p < 0.05) in both TLR1 and TLR2, which indicates an excess of intermediate frequency alleles and may suggest balancing selection. In both loci, the regions with D > 0 were located within LRR (Fig. 1). LRRs form horseshoe structures in the extracellular domain of the TLR molecules, which are crucial for the TLR function, as they form sites involved in binding with pathogen-derived motifs or with other components of the immune system interacting with TLRs. Sites with positive D were also found within LRRs in both TLR5 and TLR6 (Fig. S1) but at 0.05 < p < 0.01. The D statistic was not significant in all sites with D < 0.

### TLR variability and susceptibility to infection

Linear modelling revealed several associations between risk of infection and presence of the most frequent amino-acid alleles (Table S6), yet after controlling for multiple comparisons, only the effects of the TLR1*aa10 and TLR5*aa02 on infection with the blood pathogen *Bartonella* remained significant or marginally significant (Table 4). Animals with the allele TLR1*aa10 were three times more often infected with *Bartonella* than those without it (adjusted p = 0.023, Table 4). Interestingly, animals with TLR1*aa10 were five times less often infected with the nematode *A. tetraptera* than those without the allele (5.8% vs. 27.7%, p = 0.019), yet after FDR correction this effect appeared not to be significant (adjusted p = 0.2, Table S6). The presence of the TLR5*aa02 was associated with increased susceptibility to *Bartonella* (adjusted p = 0.023): 41.2% animals with the allele were infected, compared to 26.8% infection among voles without it. None of the SNPs had a significant effect on the risk of infection after controlling for multiple comparisons (Table S7).

**Table 4:** Genetic terms significantly associated with a risk of infection.

genetic term	pathogen	R2	χ^2^	p	FDR	% infected	
						with	without
TLR1*aa10	*Bartonella*	0.144	10.6169	0.001	0.024	11.7	33.3
TLR5*aa02	*Bartonella*	0.094	7.4701	0.006	0.066	41.2	26.8

R2 - coefficient of determination, p – p-value as fitted in a model, χ^2^ -test statistics, p – p-value, FDR – adjusted p-value using false discovery rate, % infected with and without the significant genetic variant.

## Discussion

We have examined patterns of parasite-driven selection in the TLR gene family using a free-living rodent as a study system. By combining neutrality tests with statistical models we provide the first evidence for balancing selection acting on TLR1 and TLR5, which present bacterial PAMPs, whereas in TLR7 and TLR9, which recognize virus-derived motifs, we found weak evidence of negative and positive directional selection, respectively.

### Selection acting on TLRs presenting bacterial PAMPs

We found no uniform pattern of selection across TLRs presenting bacterial PAMPs (TLR1, 2, 4, 5, 6). TLR4 showed significant deviation from the Hardy-Weinberg equilibrium, which resulted from an excess of heterozygotes at the site Pilchy, yet in this locus we found no other evidence for selection. In the remaining TLRs, except for TLR6, we found significant deviations from neutrality, as indicated by Fu and Li’s D* test for TLR2 and TLR5, and increased Tajima’s D in parts of TLR1 and TLR2. Although, overall, Tajima’s D was not significant, high significant values in parts of the molecules putatively interacting with PAMPs suggest parasite-driven selection. Positive D or D* values are interpreted as a signature of balancing selection but they may also arise from demographic processes such as changes in population size. However, if demographic processes had affected the frequency of alleles in the studied populations, this should be similar in all loci, yet we found no consistent pattern over the genes studied^50^. Similarly, no uniform pattern arose from F_st_ analysis. In TLR1 and TLR5, F_st_ was close to 0.1, indicating moderate population structure, while in TLR2, 4 and 6 it was lower or similar to the value obtained from neutral markers, which was 0.072^19^. In Sweden, a somewhat similar discrepancy between population structure in TLR2 and in neutral loci observed in bank voles was interpreted as a evidence for local parasite-driven selection acting on this locus^51^. Thus, the observed differences between our sites in Poland in the TLR allele frequencies are more likely to result from selection than from demographic processes.

Macro evolutionary studies of TLR evolution have underlined the role of directional selection, either positive^8,10^, purifying^11,13^ or both^12^. However, within a species the results have often been inconsistent: for instance in humans, the European population showed signatures of balancing selection^15^, whereas the Indian population had notably higher number of non-synonymous substitutions compared to other studied areas^52,53^.

Except for the human study cited above, our work is the first to demonstrate balancing selection acting on TLR genes in mammals. Parasite-driven balancing selection, either based on frequency dependent selection or rare allele advantage, has been well described in the case of MHC genes (see the work reviewed in^39^), although its importance for innate immunity genes has yet to be explained. To date, evidence for balancing selection has been found in cytokines IL1b, IL 2 and TNF α in field voles, as indicated by significant deviation from neutrality in one (IL1b, IL 2) or several tests (TNF α)^43,50^. In the current study, we found similarly high heterozygosity (>0.7) in TLR1, TLR2 and TLR5, and significant results of Fu and Li’s D* test for TLR2 and TLR5, and positive values of Tajima’s D in LRRs in TLR1 and TLR2. Together with significant association between certain variants of TLR1 and TLR5 and susceptibility to pathogens, our results indicate balancing selection acting on these loci.

Differences in pathogens community between populations may explain why it is difficult to detect balancing selection in a macro evolutionary scale. On a large scale the populations are likely to be affected by different pathogen species, whereas on a small scale they are likely to share similar species differing in subtler ways, for instance in their antigenicity. Indeed, compared to directional selection reflecting large-scale differences, the effect of balancing selection is often subtle^16^ and seldom persists over a long time^17^. However, as indicated in our study, it seems to be an important factor driving evolution of MHC genes in a micro evolutionary scale.

Directional selection observed within TLRs has been often ascribed to the conserved structure of the PAMPs^54^ but these pathogen-derived motifs are not invariable. For instance, TLR4 recognizes lipid A, a conserved part of lipopolysacharides (LPS) forming the cell wall of Gram negative bacteria. The outer part of the LPS is highly variable, and its polymorphism differentiates strains into particular serotypes^55^. What is more, some pathogens can modify their lipid A to avoid recognition by TLR4, which gives an opportunity for parasite-driven selection^56^.

TLR5, in which we found signatures of balancing selection, recognizes bacterial flagellin, a molecule that is crucial for pathogen motility. The N- and C-terminal parts of the flagellin are conserved but the central region of the protein, which has antigenic properties, is highly variable both within and among bacteria species^57,58^. We found significant associations between allele TLR5*02 and susceptibility to infection with *Bartonella sp*., a flagellate bacteria. Samples collected from rodents living in our study area revealed that *Bartonella* strains are highly recombinant^59^, and some of them displayed high variability in virulence gene VirB5^60^. Such a variability gives an opportunity for a micro evolutionary interplay between bacterial variants and TLR5 alleles, in a similar manner as in the case of MHC genes^39^, which might explain the observed signatures of balancing selection in this locus.

### TLR polymorphism and susceptibility to infections

Functional links between genetic variants and susceptibility are crucial for determining recent or ongoing selection processes^50^, as the ability of a host to resist infection is expected to have a direct impact on its fitness. Except for association studies in humans and livestock, evidence from free-living species is rare and no previous study has considered several TLR loci. In bank voles, TLR2 haplotypes associated with infection with *Borrelia* were identified^7^, and an association between TLR2 and cestode burden was observed in field voles^43^. Here, we found a significant effect of TLR1 and TLR5 alleles on the risk of infection with *Bartonella sp*.

TLR1 presents bacterial lipoproteins, and together with TLR2 and TLR6 they act as a functional subgroup whose members interact with each other, forming heterodimers^61^. Thus, although none of the TLR2 and TLR6 variants remained significant after correcting for multiple comparisons, we may interpret the associations involving TLR1 as a functional outcome of the polymorphism within the TLR1-2-6 group, which underlies the complexity of the associations between components of the innate immunity.

The allele TLR5*02 increased the risk of infection with *Bartonella*. The presence of alleles associated with increased susceptibility has been widely discussed in the case of MHC, and several explanations have been proposed. Under balancing selection, pathogens are likely to adapt to the most frequent allele, and the rare variants are expected to be associated with resistance^62^. However, the process of removing “susceptible” alleles from a population is slow^63^. Alternatively, the presence of a “susceptible” allele in a population may result from the fact that individuals without this variant did not survive an infection and thus could not be sampled, while hosts with the allele persisted in a population despite heavy pathogen load^64^.

The pathogen community within a host is relatively stable, and the presence of one species may alter the risk of infection with another^65,66^. For instance, in field voles, the risk of infection with *Babesia* increased when an animal was already infected with *Anaplasma*, but voles infected with *Babesia* were more resistant t*o Bartonella*^67^. On a molecular level, these associations involve interactions between different pathogen species, and between pathogens and the host immune system. For instance, nematodes may modulate host immune response so that it affects potentially competing species^66^ or larval stages of their own species in a process called concomitant immunity^68^, and negative associations between nematode species infecting free living rodents have been reported^18,66^. Microbial pathogens may evade the immune response by modifying their surface proteins interacting with components of the host immune system or through interference with downstream signalling pathways^55^. Moreover, PAMPs derived from a single pathogen are often recognized by various TLRs^69^, and activation of a given TLR by one pathogen may alter its reactivity to another^70^. Thus, the association between the presence of TLR5*aa02 and susceptibility to infection with *Bartonella* reported in the current paper might result from interactions of this allele with other components of the immune system and/or other pathogens, which were either not recognized in the current study or were too rare to be included in the models.

Another factor that might influence susceptibility is previous exposure to the pathogen. The population studied in the current paper was sampled when the prevalence of *Bartonella* was the highest^71^. Given the relatively short lifespan of a vole (3–4 months) and seeing as voles usually get infected when several weeks old and the infection lasts 1.5 months on average^71^, we may assume that among the individuals classified as uninfected in the current study, only a few were previously exposed to the pathogen and had had enough time to clear from infection.

In the current paper we found weak evidence for the role of TLRs in immunity against nematodes. The presence of the allele TLR1*aa10 decreased the risk of infection with the nematode *A. tetraptera* by 5-fold, yet this effect was insignificant after correcting for multiple comparisons. Although TLRs are not capable of recognizing motifs derived from multicellular pathogens, they may contribute to the immune response against nematodes through interaction with their *Wolbachia*-like bacterial endosymbionts^72,73^, and associations between TLR2 variants and susceptibility to cestodes was found in common voles^43^. Moreover, helminth infections limit the density of red blood cell dwelling microparasites through anemia and suppression of an inflammatory Th-1 type immune response^74^. Taken together, although components of the innate immunity do not primary interact with multicellular pathogens, the presence of helminth infections may affect evolution of the TLR genes and should be considered in future studies.

Although associations between single-nucleotide changes in TLR genes and diseases were detected in human studies (e.g.^6^), we did not find an effect of any SNP on the risk of infection. In free-living species, genotype at several SNPs within cytokines altered the risk of infection^43^. Contrary to the current study, where we covered 47–99% of the coding sequences, study^43^ genotyped only a few SNPs per locus. Thus, these might not reflect the overall polymorphism. In our work, haplotypes usually differed from each other in several positions, which suggests that the effect of a single substitution in a protein composed of several hundreds of amino-acids may be undetectable. Moreover, the significant effects of haplotypes underlie the functional importance of the combination of SNPs within a sequence.

### Selection acting on viral TLRs

In the present study we were not able to test for associations between TLR7 or TLR9 and susceptibility to infection due to low variability at these loci, thus we were able to analyse the selection pattern acting on these loci only based on the results of neutrality tests.

TLR7 recognizes ssRNA from viruses, whereas TLR9 binds CpG DNA from viruses but also bacteria. We found differences in the polymorphisms of TLR7 and TLR9 compared to the remaining loci involved in the response against bacterial or protozoan pathogens. TLR7 was characterized by a low number of segregating sites, low haplotype diversity, and consistent excess of homozygotes in both studied sites. This characteristic, together with negative Tajima’s D and neutrality index NI > 1 (albeit with both values not significant), may suggest purifying selection. TLR9 also had a low number of segregating sites and low haplotype diversity but significant NI < 1 suggested positive selection. In both loci we observed high and significant F_st_ values, indicating population differentiation (F_st_ = 0.11 in TLR7 and 0.38 in TLR9). However, the results of neutrality tests may be affected by a low overall variability in these loci and high differences in allele frequencies between sites, thus they should be interpreted with caution.

Differences in both the strength and direction of selection acting on TLRs presenting virus-derived nucleic acids compared to TLR loci presenting bacterial PAMPs have previously been reported^8,14^, and the low polymorphism of TLR7 has been attributed to low diversifying selective pressures acting on viral ssRNA^14^. In an alternative explanation^8^, prevention of self-reactivity has been proposed as the main reason for low polymorphism within TLRs interacting with viral PAMPs: these TLRs are expressed in the endoplasmatic reticulum, so there is a risk of an immune response against self nucleic acids.

TLR3, 7, and 9 are not the only pattern recognition receptors that interact with viruses. Viruses that have already entered into the cytoplasm may be detected by RIG-I-like receptors (RLRs), such as RIG-I and MDA5, which contain a helicase domain capable of binding to viral RNA. Their activation induces pro-inflammatory innate immune response, and each RLR recognize different viral patterns^75^. Several viral proteins that inhibit RLRs signaling have been identified suggesting that pathogens evolve to avoid recognition by RLR-induced pathway^75^. Although there is no studies comparing selective pressure acting on both classes of these viral-sensing PRRs, we may hypothesise that strong selective pressure on RIG-I and MDA5 may weaken selection acting on TLR7 or TLR9. Moreover, nucleic acids are not the only viral patterns recognized by components of the innate immunity. For instance, highly variable proteins expressed on a viral envelope are recognized by TLR2 and TLR4^69^, what may also contribute to the relaxed selection acting on TLR7 and TLR9.

On the other hand, low polymorphism of TLR9 may contribute to the biochemical properties of the molecule, which undergoes considerable conformational change after binding a ligand^76^. To preserve this ability, a large part of the sequence has to be conserved. Hence, the polymorphism in TLR9 may be explained by mechanisms maintaining the stereochemical properties of the molecule^61^, interactions with other components of the immune system, or preventing self-reactivity, rather than selective pressure from pathogens. Testing this hypothesis will require further studies using laboratory animals, including infections with RNA and DNA viruses.

## Electronic supplementary material

Supplementary materials
