# Author Correction: Signatures of balancing selection in toll-like receptor (TLRs) genes – novel insights from a free-living rodent

**DOI:** 10.1038/s41598-019-43510-1

**Published:** 2019-05-09

**Authors:** Agnieszka Kloch, Marius A. Wenzel, Dominik R. Laetsch, Olek Michalski, Anna Bajer, Jerzy M. Behnke, Renata Welc-Falęciak, Stuart B. Piertney

**Affiliations:** 1 University of Warsaw, Department of Ecology, Faculty of Biology, Biological and Chemical Research Centre, ul. Zwirki i Wigury 101, 02-089 Warszawa, Poland; 2 University of Aberdeen, Institute of Biological and Environmental Sciences, Tillydrone Avenue, Aberdeen, AB24 2TZ UK; 3 The James Hutton Institute, Dundee, DD2 5DA United Kingdom; 4 University of Edinburgh, Institute of Evolutionary Biology, Edinburgh, EH9 3JT UK; 5 Data Analysis & Visualisation, ul. Przy Agorze 5a m. 98, 01-960 Warszawa, Poland; 6 University of Warsaw, Department of Parasitology, Faculty of Biology, ul. Miecznikowa 1, 02-096 Warszawa, Poland; 7 School of Life Sciences, University of Nottingham, Nottinghamshire, NG7 United Kingdom

Correction to: *Scientific Reports* 10.1038/s41598-018-26672-2, published online 30 May 2018

Anna Bajer and Jerzy M. Behnke were omitted from the author list in the original version of this Article.

The Author Contributions section now reads:

“A.K. conceived, designed and performed the study, analysed data, and wrote the paper with contributions from D.R.L., M.A.W. and S.B.P. D.R.L. analysed genetic data; O.M. contributed to the data analysis and wrote scripts; R.W.F. analysed infection with blood pathogens. J.M.B. conceived the main schedule of the parasitological study, participated in the field work and contributed to the parasite identification. A.B. planned, supervised and participated in the field work, collected the samples and performed primary evaluation of parasite material.”

The Acknowledgements section now reads:

“The work was supported by grant no. DEC-2012/07/B/NZ8/00058 from the Polish National Science Centre to A.K. Field studies were funded by grant MNiI 2P04C09827 „Badania naturalnych źródeł zarażenia mikropasożytów patogennych dla człowieka” to AB. We are thankful to Dr. hab W. Babik who provided access to an Illumina MiSeq platform, and to K. Dudek who prepared the Nextera library. Special thanks to A. Biedrzycka for her valuable comments on the final version of the manuscript. We also would like to thank two anonymous reviewers for their valuable comments that helped to improve the manuscript.”

In addition, the legend of Figure 1 was incorrect,

“Sliding window Tajima’s D for TLR1 and TLR1. Stars indicate regions where D was significant at p < 0.05. Grey bars below the graph represent location of LRRs.”

now reads:

“Sliding window Tajima’s D for TLR1 and TLR2. Stars indicate regions where D was significant at p < 0.05. Grey bars below the graph represent location of LRRs.”

Furthermore, this Article contained an error in the Methods section under the subheading ‘Data Accessibility’,

“Raw reads have been stored in SRA archive, biosample no. 7414489. Haplotypes are stored in GenBank (Access nos. MF471907 – MF472008). Should the manuscript be accepted, the data on infection status and genotypes of individuals will be archived in Dryad and the data DOI will be included at the end of the article.”

now reads:

“Raw reads have been stored in SRA archive, biosample no. 7414489. Haplotypes are stored in GenBank (Access nos. MF471907 – MF472008). Data on infection status and genotypes of individuals are available at github.com/drowca/TLRs”

These errors have now been corrected in the PDF and HTML versions of the Article, and in the accompanying Supplementary materials.

