# Supplementary material for: Signatures of balancing selection in toll-like receptor (TLRs) genes – novel insights from a free-living rodent
Source: Sci Rep. 2018 May 30;8:8361. doi: 10.1038/s41598-018-26672-2 (PMC5976762; doi:10.1038/s41598-018-26672-2)
Supplement: Supplementary file 1 — Supplementary materials [file 41598_2018_26672_MOESM1_ESM.pdf]

SUPPLEMENTARY MATERIALS

Signatures of balancing selection in toll-like receptor (TLRs) genes – novel insights from a free-living rodent

Agnieszka Kloch[1], Marius A. Wenzel[2], Dominik R. Laetsch[3,4], Olek Michalski[5], Anna Bajer[6], Jerzy M Behnke[7], Renata Welc-Falęciak[6], and Stuart B. Piertney[2]

1 University of Warsaw, Department of Ecology, Faculty of Biology, Biological and Chemical Research Centre, ul. Zwirki i Wigury 101, 02-089 Warszawa, Poland

2 University of Aberdeen, Institute of Biological and Environmental Sciences, Tillydrone Avenue, Aberdeen AB24 2TZ, UK

3 The James Hutton Institute, Dundee DD2 5DA, United Kingdom

4 University of Edinburgh, Institute of Evolutionary Biology, Edinburgh EH9 3JT, United Kingdom

5 perlism.org, Data Analysis & Visualisation, ul. Przy Agorze 5a m. 98, 01-960 Warszawa, Poland

6 University of Warsaw, Department of Parasitology, ul. Miecznikowa 1, 02-096, Warszawa, Poland

7 School of Life Sciences, University of Nottingham, Nottinghamshire NG7, United Kingdom

SUPPLEMENTARY TABLES

Table S1. Sequences of primers and PCR conditions used to identify infections with blood parasites. Tm – melting temperature of the primers .

parasite	gene	primer	Sequence 5' → 3'	Tm	Reference
Bartonella sp.	gltA	BhCS.781p<br>BhCS.1137n	GGGGACCAGCTCATGGTGG<br>AATGCAAAAAGAACAGTAAACA	51	Norman et al. 1995
Babesia microti	18S rRNA	CRYPTO R<br>CRYPTO F	GAATGATCCTTCCGCAGGTTCACCTAC<br>AACCTGGTTGATCCTGCCAGTAGTCAT	63	Herwaldt et al. 2003
	18S rRNA	Bab1<br>Bab4	CTTAGTATAAGCATACAGC<br>ATAGGTCAGAAACTTGAATGATACA	55	Persing et al.. 1992

Table S2. Differences between sites in parasite prevalence (percentage of infected hosts) following Kloch et al. (2010).

	% infected		differences between sites	
	Urwitałt	Pilchy	χ^2	p
Nematodes				
Heligmosomum mixtum	79.1	0	51.231	<0.001
Heligmosomoides glareoli	0	46.3	23.172	<0.001
Aspiculuris tetraptera	9.3	41.4	9.926	0.002
Intestinal protoza				
Cryptosporium sp.	34.1	76.9	11.364	<0.001
Blood parasites				
Haemobartonella sp.	51.2	58.9	0.2378	0.626
Hepatozoon sp.	28.2	27.0	0.0001	0.999
Bartonella sp.	27.9	26.8	0.0001	0.999
Babesia sp.	23.25	17.1	0.1877	0.665
Trypanosoma sp.	18.6	12.8	0.1709	0.679

Table S3. Sequences of primers and PCR conditions used to amplify TLR genes of the innate immune response in the bank vole. Tm – melting temperature of the primers, t – extension time in seconds, cycles – number of cycles in the PCR reaction. PCR program consisted of denaturation at 98 ºC for 10 sec, annealing for 30 sec at Tm, and extension at 72ºC (time varied), followed by final extension for 10 min at 72ºC.

locus	primer sequence 5'→3'	Tm	t	cycles
TLR1	AGCTGAGGGTCYTGATRATGT	62	120	35
	CCAGCAAGATWAGGATTAAG			
TLB[1]	CGTGTTCTGTGGACCTTGTG	65	90	35
	CTAACATCCAGCACCTCCAG			
	CTTGACATCAGCCGGAACAG	63	120	35
	GAACCTAGGACTTTATTGCAGTTCTC			
TLR4	CCAGGTGTGAAATTGAGACA	59	120	35
	CTTCTCCAGAAGATGTGCCTC			
TLR5	CTCAGCTTCAACTACATCAG	60	120	35
	CCCTCTGATrGTCTCATGTC			
TLR6	CAAACAGGAACCTTACTCATGTC	64	100	35
	CCCACGTTTGCCCTTCTCAGTAG			
TLR7	CTTAGTTTCTASAGTCCTTG	59	120	35
	CGGAGCTGAAGAAACTTAGA			
TLR9	CTTCCTACCCTGTGAGCTGAAG	65	150	30
	GGTTATAGAAGTGGCGGTTGTCC			

[1] primers by Tschirren et al. (2011)

S4. Results of GLMs testing for an effect of non-genetic terms (host body mass. host sex. and site) on parasite prevalence. χ^2 – chi-squared test statistic. p – p-value. df- degrees of freedom. *H. mixtum* and *H. glareoli* were analysed in single sites (Urwitałt and Pilchy respectively). as each species was observed only in one site.

parasite	effect	χ^2	df	p
H. mixtum	host sex	0.003	1	0.961
	host body mass	3.2800	1	0.071
H. glareoli	**host sex**	**8.949**	**1**	**0.003**
	host body mass	2.393	1	0.122
A. tetraptera	**site**	**18.511**	**1**	**<0.001**
	host sex	1.655	1	0.198
	**host body mass**	**4.317**	**1**	**0.038**
Cryptosporidium	**site**	**11.674**	**1**	**<0.001**
	host sex	1.107	1	0.292
	**host body mass**	**4.082**	**1**	**0.043**
Haemobartonella	site	0.580	1	0.446
	host sex	1.799	1	0.180
	host body mass	1.498	1	0.221
Bartonella sp.	site	0.128	1	0.720
	host sex	0.023	1	0.883
	host body mass	0.499	1	0.480
Babesia sp.	site	0.320	1	0.571
	host sex	2.927	1	0.087
	host body mass	0.336	1	0.562
Hepatozoon sp.	site	0.004	1	0.944
	host sex	0.0805	1	0.776
	host body mass	2.734	1	0.098

Table S5. Results of Fu and Li D* neutrality test with a mouse sequence as an outgroup.

	Fu & Li D*
TLR1	0.270^{ns}
TLR2	**2.171**$^{<0.02}$
TLR4	-0.143^{ns}
TLR5	**1.845**$^{<0.02}$
TLR6	1.059^{ns}
TLR7	-0.143^{ns}
TLR9	0.478^{ns}

Table S6. Effect of TLR amino-acid haplotypes on a risk of infection. Each model contained as explanatory variables: genetic terms (presence/absence of amino-acid haplotypes), first and second principal component (PC1 and PC2) summarizing relatedness computed using microsatellite variation, and optional non-genetic terms as explained in *Methods*. Tables are organized by dependent variables: a) bacterial parasites, b) protists, c) nematodes. B – effect coefficient. χ^2- chi-square statistic. p – p value as estimated in a model. FDR - p-value adjusted for multiple comparisons using false discovery rate method. Values significant after correction are marked in bold.

a)	*Haemobartonella sp.*					*Bartonella sp.*					
predictors	B	R2	χ^2	p	FDR	predictors	B	R2	χ^2	p	FDR
TLR 1											
PC1	-0.199	0.002	0.244	0.622	0.622	PC1	-1.249	0.087	6.532	0.011	0.011
PC2	-0.057	0.000	0.017	0.897	0.897	PC2	0.238	-0.001	0.203	0.652	0.652
TLR1*aa01	-0.062	0.000	0.011	0.916	0.963	TLR1*aa01	-1.328	0.046	3.952	0.047	0.140
TLR1*aa02	0.217	0.002	0.186	0.666	0.963	TLR1*aa02	-0.967	0.027	2.495	0.114	0.240
TLR1*aa04	-0.394	0.003	0.284	0.594	0.963	TLR1*aa04	-1.855	0.076	4.815	0.028	0.118
TLR1*aa06	-1.667	0.079	6.881	0.009	0.183	TLR1*aa06	1.605	0.074	5.390	0.020	0.118
TLR1*aa10	-0.506	0.007	0.560	0.454	0.963	TLR1*aa10	-2.951	0.144	10.617	0.001	**0.024**
TLR 2											
PC1	-0.203	0.005	0.474	0.491	0.491	PC1	-0.384	0.019	1.300	0.254	0.254
PC2	0.010	0.000	0.001	0.980	0.980	PC2	-0.085	0.000	0.035	0.851	0.851
TLR2*aa01	-0.052	0.000	0.009	0.924	0.963	TLR2*aa01	-0.451	0.006	0.568	0.451	0.592
TLR2*aa06	0.052	0.000	0.007	0.935	0.963	TLR2*aa06	-0.039	0.000	0.003	0.959	0.959
TLR2*aa12	0.640	0.008	0.767	0.381	0.963	TLR2*aa12	0.890	0.021	1.521	0.217	0.380
TLR 4											
PC1	-0.353	0.024	1.652	0.199	0.199	PC1	-0.457	0.027	2.002	0.157	0.157
PC2	-0.085	0.001	0.043	0.835	0.835	PC2	-0.316	0.008	0.492	0.483	0.483
TLR4*aa01	0.025	0.000	0.002	0.963	0.963	TLR4*aa01	0.550	0.009	0.783	0.376	0.564
TLR4*aa02	-0.512	0.012	0.741	0.389	0.963	TLR4*aa02	-0.886	0.019	1.612	0.204	0.380
TLR4*aa03	1.248	0.028	2.046	0.153	0.963	TLR4*aa03	-16.577	0.039	4.860	0.027	0.118
TLR4*aa04	-0.102	0.001	0.038	0.845	0.963	TLR4*aa04	-0.462	0.007	0.636	0.425	0.592
TLR 5											

PC1	-0.169	0.004	0.337	0.562	0.562	PC1	-0.930	0.085	6.453	0.011	0.011
PC2	0.048	0.000	0.012	0.911	0.911	PC2	-0.705	0.028	1.926	0.165	0.165
TLR5*aa01	-0.037	0.000	0.004	0.951	0.963	TLR5*aa01	1.602	0.046	4.217	0.040	0.140
TLR5*aa02	-0.189	0.001	0.082	0.774	0.963	TLR5*aa02	2.247	0.094	7.470	0.006	**0.066**
TLR5*aa03	0.461	0.007	0.631	0.427	0.963	TLR5*aa03	1.388	0.060	3.661	0.056	0.146
TLR5*aa04	0.556	0.010	0.855	0.355	0.963	TLR5*aa04	-0.698	0.004	0.792	0.373	0.564
TLR5*aa06	0.141	0.001	0.059	0.808	0.963	TLR5*aa06	1.111	0.040	2.585	0.108	0.240
TLR 6											
PC1	-0.220	0.006	0.543	0.461	0.461	PC1	-0.406	0.020	1.519	0.218	0.218
PC2	-0.249	0.004	0.360	0.549	0.549	PC2	-0.144	0.001	0.102	0.750	0.750
TLR6*aa01	0.031	0.000	0.003	0.955	0.963	TLR6*aa01	-0.200	0.003	0.109	0.741	0.864
TLR6*aa02	0.101	0.000	0.026	0.871	0.963	TLR6*aa02	-0.102	0.000	0.020	0.887	0.931
TLR6*aa04	0.781	0.025	2.127	0.145	0.963	TLR6*aa04	-0.374	0.006	0.414	0.520	0.642
TLR6*aa05	0.709	0.013	1.173	0.279	0.963	TLR6*aa05	-0.110	0.000	0.027	0.870	0.931

b)			Cryptosporidium sp.						Babesia sp.						Hepatozoon sp.		
predictors	B	R2	χ^2	p	FDR	predictors	B	R2	χ^2	p	FDR	predictors	B	R2	χ^2	p	FDR
								TLR 1									
mass	-0.125	0.039	3.687	0.055	0.055	PC1	-0.254	0.004	0.269	0.604	0.604	PC1	-0.426	0.014	1.054	0.305	0.305
PC1	-0.512	0.009	0.595	0.441	0.441	PC2	-0.531	0.011	1.025	0.311	0.311	PC2	0.106	0.000	0.043	0.836	0.836
PC2	0.184	0.002	0.134	0.715	0.715	TLR1*aa01	-0.014	0.000	0.000	0.986	0.991	TLR1*aa01	0.235	0.002	0.148	0.701	0.854
site	-1.653	0.017	1.882	0.170	0.170	TLR1*aa02	1.284	0.061	4.529	0.033	0.269	TLR1*aa02	-0.263	0.002	0.221	0.639	0.854
TLR1*aa01	-0.162	0.000	0.059	0.808	0.989	TLR1*aa04	-0.249	0.000	0.077	0.782	0.912	TLR1*aa04	-0.707	0.012	0.871	0.351	0.705
TLR1*aa02	-1.354	0.052	5.810	0.016	0.227	TLR1*aa06	0.733	0.012	0.850	0.357	0.673	TLR1*aa06	-0.057	0.000	0.007	0.936	0.971
TLR1*aa04	-0.850	0.008	0.794	0.373	0.767	TLR1*aa10	0.010	0.000	0.000	0.991	0.991	TLR1*aa10	-0.252	0.002	0.117	0.732	0.854
TLR1*aa06	0.723	0.011	0.966	0.326	0.767												
TLR1*aa10	0.028	0.000	0.001	0.971	0.989												
								TLR 2									
mass	-0.113	0.030	3.293	0.070	0.070	PC1	-0.536	0.019	1.646	0.200	0.200	PC1	0.159	0.002	0.234	0.628	0.628
PC1	-0.535	0.009	0.693	0.405	0.405	PC2	-0.470	0.014	0.826	0.363	0.363	PC2	0.255	0.006	0.273	0.601	0.601
PC2	-0.066	0.000	0.020	0.888	0.888	TLR2*aa01	0.850	0.022	1.445	0.229	0.673	TLR2*aa01	0.073	0.001	0.013	0.908	0.971
site	-0.455	0.002	0.172	0.678	0.678	TLR2*aa06	1.768	0.048	4.285	0.038	0.269	TLR2*aa06	-1.069	0.022	1.860	0.173	0.705
TLR2*aa01	0.095	0.000	0.026	0.872	0.989	TLR2*aa12	0.733	0.008	0.795	0.373	0.673	TLR2*aa12	0.805	0.017	1.109	0.292	0.705
TLR2*aa06	-0.881	0.014	1.403	0.236	0.767												
TLR2*aa12	-1.423	0.036	3.110	0.078	0.545												
								TLR 4									
mass	-0.106	0.037	3.106	0.078	0.078	PC1	-0.117	0.002	0.112	0.737	0.737	PC1	-0.188	0.003	0.375	0.540	0.540
PC1	-0.422	0.006	0.456	0.499	0.499	PC2	-0.331	0.003	0.476	0.490	0.490	PC2	-0.024	0.000	0.002	0.964	0.964
PC2	0.011	0.000	0.001	0.981	0.981	TLR4*aa01	0.491	0.010	0.498	0.481	0.673	TLR4*aa01	-0.482	0.009	0.628	0.428	0.705
site	-1.116	0.016	1.157	0.282	0.282	TLR4*aa02	-0.606	0.012	0.681	0.409	0.673	TLR4*aa02	-0.290	0.000	0.147	0.702	0.854
TLR4*aa01	-0.049	0.000	0.007	0.931	0.989	TLR4*aa03	-0.589	0.000	0.285	0.594	0.764	TLR4*aa03	0.748	0.006	0.688	0.407	0.705
TLR4*aa02	-0.067	0.000	0.009	0.925	0.989	TLR4*aa04	-0.962	0.025	1.972	0.160	0.673	TLR4*aa04	1.391	0.080	5.760	0.016	0.344
TLR4*aa03	0.730	0.007	0.703	0.402	0.767												
TLR4*aa04	-0.622	0.012	1.082	0.298	0.767												
								TLR 5									

mass	-0.132	0.056	4.490	0.034	0.034	PC1	-0.332	0.019	0.780	0.377	0.377	PC1	-0.244	0.006	0.509	0.475	0.475
PC1	-0.100	0.000	0.026	0.872	0.872	PC2	-0.453	0.012	0.683	0.409	0.409	PC2	-0.163	0.002	0.099	0.753	0.753
PC2	-0.026	0.000	0.003	0.958	0.958	TLR5*aa01	-0.090	0.001	0.013	0.908	0.991	TLR5*aa01	1.317	0.035	3.218	0.073	0.507
site	-2.096	0.046	3.181	0.075	0.075	TLR5*aa02	1.237	0.044	2.224	0.136	0.673	TLR5*aa02	1.571	0.038	3.775	0.052	0.507
TLR5*aa01	-0.578	0.010	0.734	0.392	0.767	TLR5*aa03	0.605	0.011	0.665	0.415	0.673	TLR5*aa03	0.413	0.006	0.324	0.569	0.854
TLR5*aa02	0.813	0.019	1.200	0.273	0.767	TLR5*aa04	0.787	0.021	1.145	0.285	0.673	TLR5*aa04	0.557	0.006	0.605	0.437	0.705
TLR5*aa03	-0.061	0.000	0.009	0.926	0.989	TLR5*aa06	-0.630	0.005	0.602	0.438	0.673	TLR5*aa06	0.822	0.016	1.452	0.228	0.705
TLR5*aa04	-0.437	0.003	0.366	0.545	0.901												
TLR5*aa06	0.314	0.001	0.239	0.625	0.937												
									TLR 6								
mass	-0.129	0.043	4.339	0.037	0.037	PC1	-0.347	0.019	0.755	0.385	0.385	PC1	-0.197	0.004	0.337	0.561	0.561
PC1	-0.198	0.003	0.090	0.764	0.764	PC2	-0.479	0.015	0.860	0.354	0.354	PC2	0.353	0.006	0.484	0.487	0.487
PC2	0.089	0.001	0.035	0.852	0.852	TLR6*aa01	0.378	0.003	0.248	0.618	0.764	TLR6*aa01	-0.603	0.011	0.900	0.343	0.705
site	-1.503	0.019	1.878	0.171	0.171	TLR6*aa02	1.635	0.060	4.409	0.036	0.269	TLR6*aa02	-0.026	0.000	0.001	0.971	0.971
TLR6*aa01	0.378	0.002	0.343	0.558	0.901	TLR6*aa04	0.532	0.012	0.524	0.469	0.673	TLR6*aa04	-1.039	0.033	2.760	0.097	0.507
						TLR6*aa05	0.716	0.014	0.808	0.369	0.673	TLR6*aa05	-0.622	0.010	0.791	0.374	0.705

c)		*Aspiculurs tetraptera*						*Heligmosomum mixtum*						*Heligmosomoides glareoli*			
								TLR 1									
predictors	B	R2	χ^2	p	FDR	predictors	B	R2	χ^2	p	FDR	predictors	B	R2	χ^2	p	FDR
mass	0.141	0.064	3.239	0.072	0.072	PC1	0.562	0.016	0.358	0.549	0.549	PC1	0.870	0.015	0.524	0.469	0.469
PC1	-0.166	0.000	0.050	0.824	0.824	PC2	-0.711	0.015	0.651	0.420	0.420	PC2	1.589	0.107	5.012	0.025	0.025
PC2	0.640	0.014	1.286	0.257	0.257	TLR1*aa01	-0.982	0.025	1.104	0.293	0.537	sex	-2.313	0.206	9.790	0.002	0.002
site	-2.885	0.066	4.171	0.041	0.041	TLR1*aa02	1.931	0.071	3.452	0.063	0.347	TLR1*aa01	-0.020	0.000	0.000	0.985	0.997
TLR1*aa01	0.442	0.007	0.374	0.541	0.962	TLR1*aa06	-0.711	0.014	0.556	0.456	0.537	TLR1*aa02	0.003	0.000	0.000	0.997	0.997
TLR1*aa02	-0.720	0.019	1.296	0.255	0.892	TLR1*aa10	-1.055	0.032	0.896	0.344	0.537	TLR1*aa04	-0.016	0.000	0.000	0.987	0.997
TLR1*aa04	-0.384	0.006	0.222	0.638	0.962							TLR1*aa06	-0.862	0.017	0.523	0.470	0.953
TLR1*aa06	0.241	0.002	0.079	0.779	0.962							TLR1*aa10	-1.234	0.032	1.124	0.289	0.953
TLR1*aa10	-2.440	0.068	5.498	0.019	0.200												
								TLR 2									
mass	0.142	0.047	3.382	0.066	0.066	PC1	1.992	0.099	3.310	0.069	0.069	PC1	0.112	0.000	0.012	0.912	0.912
PC1	-0.340	0.003	0.232	0.630	0.630	PC2	0.058	-0.001	0.004	0.952	0.952	PC2	1.236	0.079	4.125	0.042	0.042
PC2	0.371	0.005	0.534	0.465	0.465	TLR2*aa01	-1.826	0.065	2.001	0.157	0.537	sex	-2.035	0.162	7.799	0.005	0.005
site	-1.416	0.021	1.300	0.254	0.254	TLR2*aa06	-0.025	-0.001	0.001	0.982	0.982	TLR2*aa01	-0.375	0.006	0.185	0.667	0.953
TLR2*aa01	0.059	0.000	0.008	0.930	0.985	TLR2*aa12	19.248	0.101	3.692	0.055	0.347	TLR2*aa11	-0.757	0.007	0.469	0.493	0.953
TLR2*aa06	-1.631	-0.001	1.632	0.201	0.846	TLR2*aa14	17.540	0.027	1.442	0.230	0.537	TLR2*aa12	-0.267	0.001	0.041	0.840	0.997
TLR2*aa12	-0.376	0.004	0.162	0.688	0.962	TLR2*aa23	-3.425	0.158	5.063	0.024	0.347						
								TLR 4									
mass	0.120	0.052	2.769	0.096	0.096	PC1	0.444	-0.006	0.236	0.627	0.627	PC1	0.773	0.014	0.403	0.525	0.525
PC1	-0.308	0.003	0.209	0.648	0.648	PC2	-0.629	0.025	0.583	0.445	0.445	PC2	1.550	0.074	4.332	0.037	0.037
PC2	0.387	0.012	0.609	0.435	0.435	TLR4*aa01	0.686	-0.013	0.536	0.464	0.537	sex	-2.632	0.227	10.642	0.001	0.001
site	-2.009	0.036	2.952	0.086	0.086	TLR4*aa03	-1.260	0.022	1.136	0.287	0.537	TLR4*aa01	-2.328	0.139	6.079	0.014	0.274
TLR4*aa01	0.422	0.015	0.422	0.516	0.962	TLR4*aa04	-0.563	0.002	0.288	0.591	0.650	TLR4*aa02	0.713	0.024	0.618	0.432	0.953
TLR4*aa02	0.208	0.000	0.089	0.765	0.962	TLR4*aa10	-2.130	0.098	3.687	0.055	0.347	TLR4*aa04	0.614	0.002	0.336	0.562	0.953
TLR4*aa03	0.628	-0.003	0.298	0.585	0.962							TLR4*aa06	-1.005	0.021	0.787	0.375	0.953
TLR4*aa04	-0.283	-0.001	0.173	0.677	0.962												

TLR 5																	
mass	0.152	0.052	3.327	0.068	0.068	PC1	-0.274	0.001	0.090	0.764	0.764	PC1	0.784	0.010	0.438	0.508	0.508
PC1	-0.053	0.000	0.006	0.941	0.941	PC2	-1.079	0.027	1.067	0.302	0.302	PC2	1.747	0.133	6.125	0.013	0.013
PC2	-0.043	-0.001	0.006	0.937	0.937	TLR5*aa01	1.367	0.013	1.094	0.296	0.537	sex	-2.554	0.208	10.536	0.001	0.001
site	-2.948	0.038	4.201	0.040	0.040	TLR5*aa02	-0.846	0.017	0.702	0.402	0.537	TLR5*aa01	-1.592	0.050	1.970	0.160	0.945
TLR5*aa01	1.554	0.036	3.591	0.058	0.407	TLR5*aa03	-0.803	0.013	0.591	0.442	0.537	TLR5*aa03	1.912	0.040	1.779	0.182	0.945
TLR5*aa02	1.196	0.012	0.992	0.319	0.958	TLR5*aa05	-0.619	0.001	0.165	0.684	0.717	TLR5*aa04	0.396	-0.002	0.200	0.655	0.953
TLR5*aa03	1.497	0.016	2.702	0.100	0.526	TLR5*aa06	-1.061	0.020	0.894	0.344	0.537	TLR5*aa06	-0.164	0.000	0.030	0.863	0.997
TLR5*aa04	-0.297	0.000	0.167	0.683	0.962	TLR5*aa15	-1.105	0.026	0.780	0.377	0.537						
TLR5*aa06	-0.135	0.000	0.032	0.859	0.985												
TLR 6																	
mass	0.157	0.065	3.759	0.053	0.053	PC1	0.316	0.004	0.165	0.684	0.684	PC1	0.664	0.004	0.366	0.545	0.545
PC1	-0.423	0.010	0.332	0.564	0.564	PC2	-0.558	0.007	0.446	0.504	0.504	PC2	1.883	0.134	6.562	0.010	0.010
PC2	0.159	0.003	0.074	0.785	0.785	TLR6*aa01	1.062	0.025	1.508	0.219	0.537	sex	-2.495	0.206	10.226	0.001	0.001
site	-3.106	0.052	5.542	0.019	0.019	TLR6*aa02	1.088	0.039	1.466	0.226	0.537	TLR6*aa01	0.497	0.007	0.237	0.627	0.953
TLR6*aa01	-1.868	0.080	6.381	0.012	0.200	TLR6*aa04	1.007	0.027	1.369	0.242	0.537	TLR6*aa04	-1.225	0.043	1.725	0.189	0.945
TLR6*aa02	0.862	0.006	0.671	0.413	0.962							TLR6*aa05	-0.818	0.009	0.735	0.391	0.953
TLR6*aa04	0.014	0.000	0.000	0.985	0.985							TLR6*aa07	0.328	0.002	0.056	0.812	0.997
TLR6*aa05	0.039	0.000	0.003	0.958	0.985												

Table S7. FDR-adjusted p-values from a GLM models testing for effects of non-synonymous SNPs on a presence of infection with parasites.

SNP	*H. mixtum*	*H.glareoli*	*A.tetraptera*	*Cryptosporidium*	*Haemobar-tonella*	*Bartonella*	*Babesia*	*Hepatozoon*
TLR1 104	1.000	1.000	0.695	0.684	0.390	0.445	0.902	0.766
TLR1 161	0.812	1.000	0.861	0.861	0.390	0.852	0.902	0.845
TLR1 236	0.678	1.000	0.395	0.746	0.849	0.844	0.902	0.541
TLR1 283	1.000	1.000	0.663	0.870	0.908	0.852	0.902	0.766
TLR1 367	1.000	1.000	0.729	0.469	0.618	0.871	0.902	0.710
TLR1 394	1.000	1.000	0.729	0.469	0.618	0.871	0.902	0.710
TLR1 562	1.000	1.000	0.772	0.469	0.505	0.906	0.902	0.666
TLR1 602	1.000	1.000	0.695	0.684	0.390	0.445	0.902	0.766
TLR1 642	1.000	1.000	0.663	0.875	0.854	0.805	0.902	0.347
TLR1 988	0.803	1.000	0.663	0.921	0.829	0.852	0.902	0.333
TLR1 1109	1.000	1.000	0.738	0.861	0.789	0.445	0.902	0.766
TLR2 1996	1.000	1.000	0.672	0.861	0.789	0.852	0.902	0.710
TLR2 1687	0.678	1.000	0.921	0.841	0.390	0.770	0.902	0.580
TLR2 1628	1.000	1.000	0.663	0.870	0.671	0.748	0.902	0.284
TLR2 1600	0.678	1.000	0.497	0.861	0.789	0.445	0.902	0.580
TLR2 1561	1.000	1.000	0.507	0.684	0.796	0.852	0.902	0.766
TLR2 1502	1.000	1.000	0.876	0.875	0.796	0.844	0.996	0.347
TLR2 1444	0.777	1.000	0.538	0.861	0.705	0.502	0.902	0.580
TLR2 1352	0.812	1.000	0.674	0.861	0.789	0.748	0.902	0.541
TLR2 1244	0.790	1.000	0.713	0.786	0.796	0.852	0.902	0.424
TLR2 1028	1.000	1.000	0.391	0.870	0.618	0.445	0.939	0.923
TLR2 981	1.000	1.000	0.391	0.870	0.618	0.445	0.939	0.923

TLR2 833	1.000	1.000	0.663	0.861	0.789	0.995	0.902	0.182
TLR2 820	0.812	1.000	0.695	0.861	0.789	0.748	0.902	0.541
TLR2 739	1.000	1.000	0.391	0.870	0.618	0.445	0.939	0.923
TLR2 587	1.000	1.000	0.663	0.870	0.646	0.744	0.902	0.284
TLR2 554	0.329	1.000	0.225	0.861	0.854	0.844	0.902	0.766
TLR2 476	0.329	1.000	0.980	0.684	0.796	0.445	0.902	0.541
TLR2 404	0.329	1.000	0.150	0.861	0.390	0.906	0.902	0.182
TLR2 379	1.000	1.000	0.663	0.767	0.849	0.445	0.902	0.923
TLR2 365	0.329	1.000	0.150	0.861	0.390	0.906	0.902	0.182
TLR2 327	0.977	1.000	0.672	0.841	0.901	0.445	0.902	0.766
TLR2 305	0.329	1.000	0.150	0.861	0.390	0.906	0.902	0.182
TLR2 295	0.790	1.000	0.861	0.861	0.789	0.762	0.902	0.541
TLR2 293	0.329	1.000	0.150	0.861	0.390	0.906	0.902	0.182
TLR2 232	1.000	1.000	0.568	0.861	0.771	0.860	0.996	0.206
TLR2 97	1.000	1.000	0.663	0.861	0.979	0.744	0.902	0.284
TLR2 26	1.000	1.000	0.663	0.853	0.789	0.990	0.971	0.587
TLR4 1833	1.000	0.740	0.270	0.786	0.796	0.852	0.902	0.726
TLR4 1544	0.329	1.000	0.961	0.930	0.996	0.852	0.902	0.290
TLR4 1268	1.000	1.000	0.663	0.870	0.796	0.805	0.902	0.693
TLR4 1267	1.000	1.000	0.663	0.870	0.796	0.805	0.902	0.693
TLR4 1187	1.000	NA	0.672	0.861	0.849	0.684	0.902	0.580
TLR4 710	1.000	1.000	0.665	0.861	0.995	0.852	0.902	0.182
TLR4 350	1.000	1.000	0.663	0.870	0.789	0.852	0.902	0.284
TLR4 157	1.000	1.000	0.663	0.870	0.789	0.852	0.902	0.284

TLR5 175	1.000	1.000	0.663	0.870	0.849	0.595	0.902	0.766
TLR5 317	1.000	1.000	0.758	0.861	0.796	0.852	0.902	0.182
TLR5 358	0.812	1.000	0.663	0.861	0.513	0.714	0.902	0.713
TLR5 404	0.925	1.000	0.889	0.684	0.849	0.684	0.902	0.923
TLR5 584	0.329	1.000	0.663	0.861	0.796	0.445	0.902	0.766
TLR5 1057	0.329	1.000	0.663	0.861	0.796	0.445	0.902	0.766
TLR5 1058	0.329	1.000	0.663	0.861	0.796	0.445	0.902	0.766
TLR5 1059	0.329	1.000	0.663	0.861	0.796	0.445	0.902	0.766
TLR5 1061	0.329	1.000	0.663	0.861	0.796	0.445	0.902	0.766
TLR5 1072	1.000	1.000	0.270	0.870	0.789	0.445	0.902	0.927
TLR5 1106	0.329	1.000	0.663	0.861	0.796	0.445	0.902	0.766
TLR5 1349	0.329	1.000	0.663	0.861	0.796	0.445	0.902	0.766
TLR5 1409	0.329	1.000	0.663	0.861	0.796	0.445	0.902	0.766
TLR5 1663	0.329	1.000	0.663	0.861	0.796	0.445	0.902	0.766
TLR5 1729	0.329	1.000	0.663	0.861	0.796	0.445	0.902	0.766
TLR5 1795	0.329	1.000	0.663	0.861	0.796	0.445	0.902	0.766
TLR5 1898	0.329	1.000	0.758	0.870	0.796	0.445	0.953	0.923
TLR5 2203	1.000	1.000	0.719	0.684	0.878	0.445	0.902	0.541
TLR5 2206	0.329	1.000	0.663	0.861	0.789	0.852	0.902	0.347
TLR5 2231	1.000	1.000	0.719	0.684	0.878	0.445	0.902	0.541
TLR6 98	1.000	0.777	0.270	0.921	0.854	0.445	0.902	0.580
TLR6 106	1.000	1.000	0.391	0.930	0.789	0.770	0.902	0.541
TLR6 181	0.977	1.000	0.270	0.786	0.390	0.852	0.902	0.923
TLR6 238	0.775	1.000	0.663	0.684	0.849	0.852	0.902	0.667

TLR6 680	0.329	1.000	0.731	0.684	0.849	0.445	0.902	0.766
TLR6 868	0.775	1.000	0.663	0.555	0.854	0.852	0.902	0.766
TLR6 898	1.000	1.000	0.270	0.684	0.390	0.852	0.902	0.923
TLR6 1195	0.416	1.000	0.944	0.469	0.789	0.981	0.902	0.927
TLR7 2270	1.000	1.000	0.663	0.684	0.942	0.445	0.902	0.284

SUPPLEMENTARY FIGURES

Fig S1. Sliding window Tajima's D for TLR5 and TLR6. Stars indicate regions where D was significant at p<0.1 Gray bars below the graph represent location of LRRs.

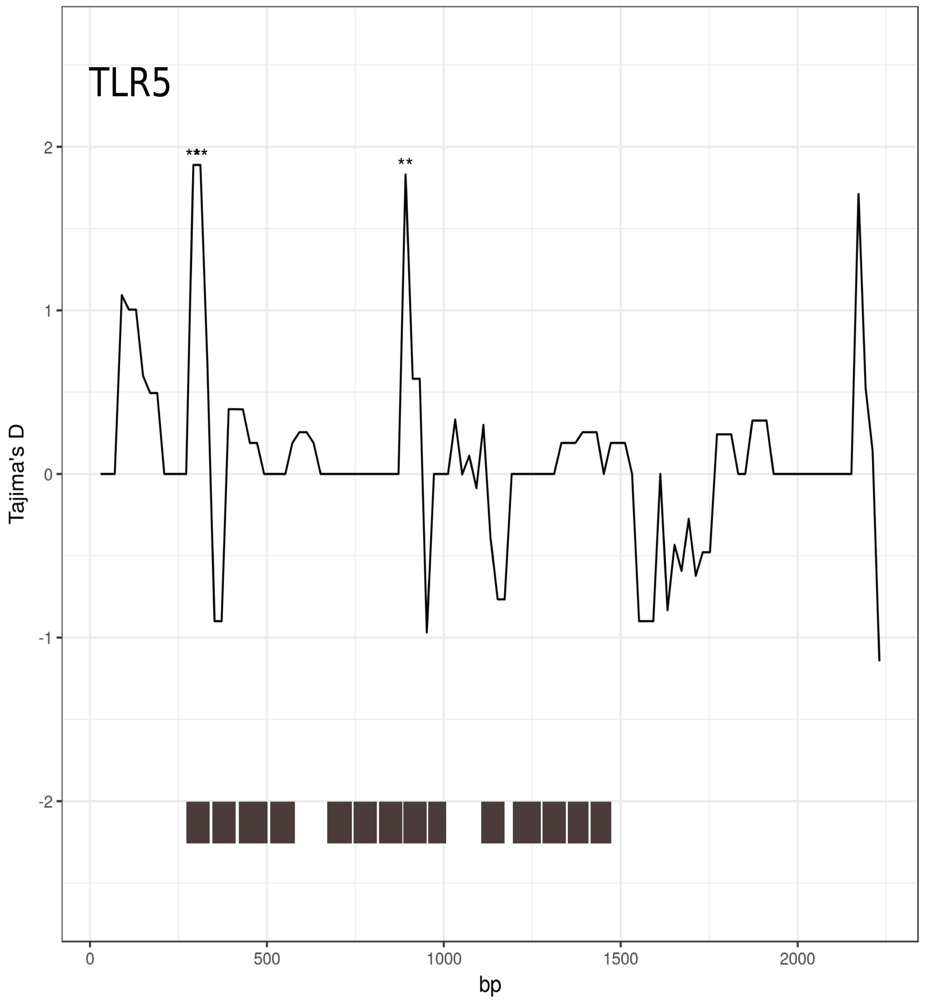

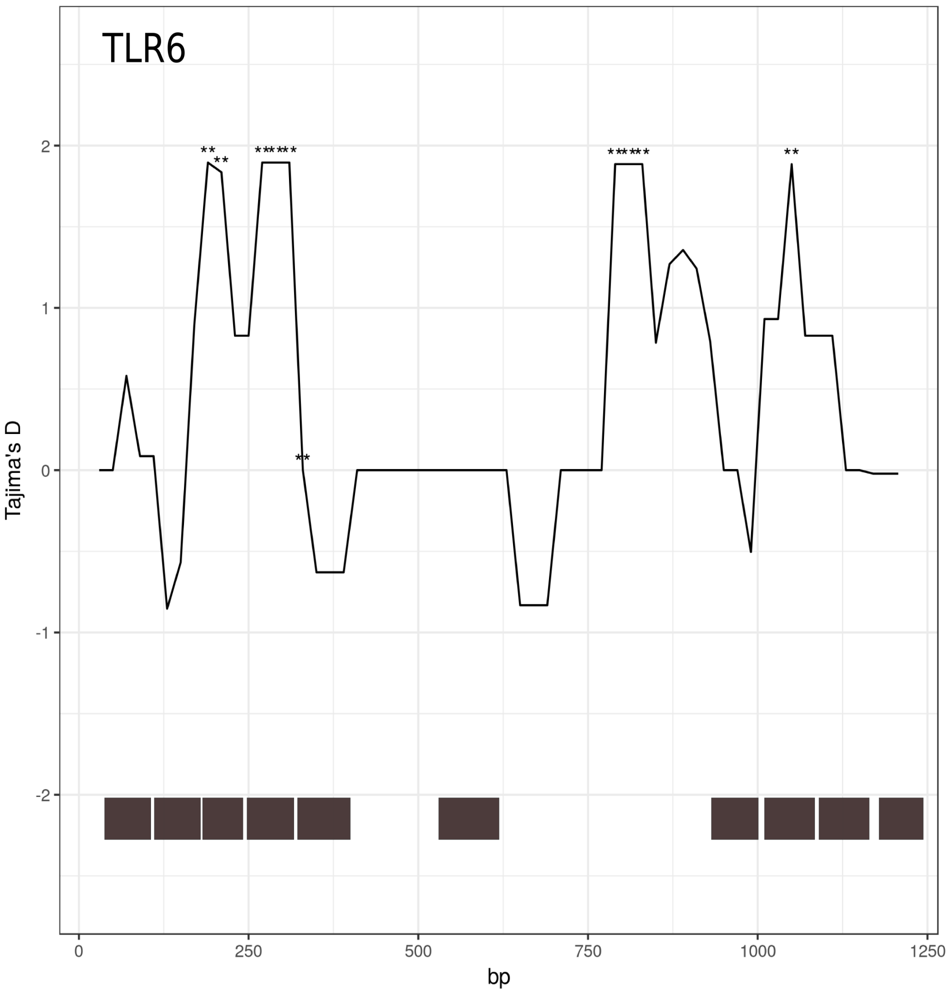

SUPPLEMENTARY FILE (SCRIPT)

Two-part python script used do phase SNPs that could not be phased based on their physical location in the reads.

This is a prototype of wrapper scripts allowing usage of PHASE to retrive haplotypes from partially phased vcf file.

The script uses Biopython to handle sequences and PyVCF for VCF format.

Usage

In the beginning the input file for PHASE is prepared from provided unphased VCF. Then one should run PHASE manually using generated file. Eventually variant sequences are produced by superimposing phased sets of variants onto reference sequence. At current stage only FASTA format is supported. All parameters are hardcoded - please edit headers of source files in order to use script s with your data.

part_1.py

```
## this part should be passed by options
chrom = 'TNFb_full'
reference_file = "data/TNFb_ref.fna"
vcffile = 'data/TNF.second.vcf'
phase_pfx = 'data/' + chrom
phase_in_fn = phase_pfx + '.inp'
phase_known_fn = phase_pfx + '.known'

locilist = dict()
genotypes = dict()
ids = set()

import vcf
vcf_reader=vcf.Reader(open(vcffile, 'r'))
for record in vcf_reader:
    if record.CHROM != chrom:
        continue
    locilist[record.POS] = 'S' if record.is_snp else 'M'
    for sample in record.samples:
        ids.add(sample.sample)
        x = sample.data.GT
        if not sample.sample in genotypes.keys():
            genotypes[sample.sample] = dict()
        if x != '.':
            genotypes[sample.sample][record.POS] = [int(a) + 1 for a in
x.split('/')]
        else:
            genotypes[sample.sample][record.POS] = ['?', '?'] if record.is_snp
else ['-1', '-1']

if not len(locilist):
    exit()

phase_in = open(phase_in_fn,  'w')
#phase_known = open(phase_known_fn,  'w')
print(len(ids),  file=phase_in)
print(len(locilist),  file=phase_in)
print("P %s" % " ".join([str(x) for x in sorted(locilist.keys())] ),
```

```
file=phase_in)
print(" ".join(locilist[y] for y in sorted(locilist.keys()) ),  file=phase_in)
for id in ids:
    print(id,  file=phase_in)
    for j in [0, 1]:
        print(" ".join(str(genotypes[id][i][j]) for i in
sorted(locilist.keys())),  file=phase_in)
phase_in.close()
```

part_2.py

```
## this part should be passed by options
chrom = 'TNFb_full'
reference_file = "data/TNFb_ref.fas"
vcffile = 'data/TNF.second.vcf'
phase_pfx = 'data/TNFb_res'
phase_in_fn = phase_pfx + '.out'
frame_positions_fn = 'data/start_stop.txt'
pos_start = 0
pos_stop = 0

refseq=''
from Bio.Seq import Seq
from Bio import SeqIO
from Bio.Alphabet import IUPAC
for seq_record in SeqIO.parse(reference_file, "fasta",  IUPAC.ambiguous_dna):
    if seq_record.id != chrom:
        continue
    refseq = seq_record

varlist = dict()
import vcf
vcf_reader=vcf.Reader(open(vcffile, 'r'))
for record in vcf_reader:
    if record.CHROM != chrom:
        continue
    varlist[record.POS] = {'alleles': record.alleles,\
                           'start': record.affected_start,\
                           'end': record.affected_end}

def read_section(filename, section):
    """Read section of file where divisions are made with lines
    BEGIN Section_Name; END Section_Name. Specifically for PHASE
    output file"""
    with open(filename) as file:
        recording = False
        for line in file:
            if "BEGIN " + section in line:
                recording = True
            elif "END " + section in line:
                recording = False
            elif recording:
                yield line

poslist = []
for line in open(phase_in_fn,  "r"):
    if line.startswith('Positions of loci'):
        tmp = line.partition(':')
```

```
        poslist = [int(x) for x in tmp[2].split()]

# TODO: this part should be made into try-except clause
if pos_start == 0 and pos_stop == 0:
    pos_stop = None

alleles = dict()
for line in read_section(phase_in_fn, 'LIST_SUMMARY'):
    a = line.split()
    allele_seq = refseq
    # WARNING: this won't work with multidigit allele symbols in PHASE output!!!
    varmap = dict(zip(poslist, ''.join(str(x) for x in a[1:-1])))
    for pos in reversed(sorted(poslist)):
        substitute = Seq(str(varlist[pos]['alleles'][int(varmap[pos])-1]),
IUPAC.ambiguous_dna)
        startN = pos - 1 if varlist[pos]['start'] == pos else varlist[pos]
['start']
        endN = varlist[pos]['end']
        allele_seq = allele_seq[:startN] + substitute + allele_seq[endN:]
    # TODO check pos_start < pos_stop in case of RC
    alleles[a[0]] = allele_seq[pos_start:pos_stop]

from Bio.SeqRecord import SeqRecord
genotypes_writeout = []
for line in read_section(phase_in_fn, 'BESTPAIRS_SUMMARY'):
    aid,  tmp,  haps = line.partition(':')
    hap1,  tmp,  hap2 = haps.lstrip(' (').rstrip(')\n').partition(',')
    genotypes_writeout.append(SeqRecord(alleles[hap1].seq,  id=aid + '-1',
description=chrom))
    genotypes_writeout.append(SeqRecord(alleles[hap2].seq,  id=aid + '-2',
description=chrom))

SeqIO.write(genotypes_writeout, chrom + ".fas", "fasta")
```
